# Predicting Species Abundance in the Face of Habitat Loss

**DOI:** 10.1371/journal.pbio.0040336

**Published:** 2006-09-26

**Authors:** Liza Gross

Habitat loss poses the greatest threat to the survival of a species, and often precipitates the demise of top predators and wide-ranging animals, like the Siberian tiger and the orangutan. Any hope of recovering such critically endangered species depends on understanding what drives changes in population size following habitat contraction.

The key question is whether population change is driven directly by changes in habitat volume, or indirectly, through responses to other species of potential predators, prey, and competitors. Ecologists rely on two types of models to predict potential responses to habitat alterations. In single-factor models, population size is controlled by one factor, such as changes in habitat size (as large blocks of forest are fragmented by clear-cutting and development, for example). This is the classic ecological model, in which habitat size drives changes in the abundance of individual species. These models also include “keystone species effects,” which look at how populations respond to the loss of a single top predator, like the tiger. In food-web models, species abundance depends on complex interactions across multiple trophic levels, including energy transfer through the food chain.

In a new study, Nicholas Gotelli and Aaron Ellison test the relative contributions of habitat contraction, keystone species effects, and food-web interactions on species abundance, and provide experimental evidence that trophic interactions exert a dominant effect. Until now, direct evidence that trophic interactions play such an important role has been lacking, in part because manipulating an intact food web has proven experimentally intractable, and in part because these different modeling frameworks have not been explicitly compared.

Gotelli and Ellison overcame such technical limitations by using the carnivorous pitcher plant *(Sarracenia purpurea)* and its associated food web as a model for studying what regulates abundance in shrinking habitats. Every year, the pitcher plant, found in bogs and swamps throughout southern Canada and the eastern United States, grows six to 12 tubular leaves that collect enough water to support an entire aquatic food web. The pitcher plant food web starts with ants, flies, and other arthropods unlucky enough to fall into its trap. Midges and sarcophagid fly larvae “shred” and chew on the hapless insect. This shredded detritus is further broken down by bacteria, which in turn are consumed by protozoa, rotifers, and mites. Pitcher plant mosquito larvae feed on bacteria, protozoa, and rotifers. Older, larger sarcophagid fly larvae also feed on rotifers as well as on younger, smaller mosquito larvae.

Working with 50 pitcher plants in a bog in Vermont, Gotelli and Ellison subjected the plants to one of five experimental treatments, in which they manipulated habitat size (by changing the volume of water in the leaves), simplified the trophic structure (by removing the top trophic level—larvae of the dipterans fly, midge, and mosquito), did some combination of the two, or none of the above (the control condition). Dipteran larvae and water were measured as each treatment was maintained; both were replaced in the control condition and more water was added in the habitat expansion treatment. These treatments mimic the kinds of changes that occur in nature as habitat area shrinks and top predators disappear from communities.

**Figure:**
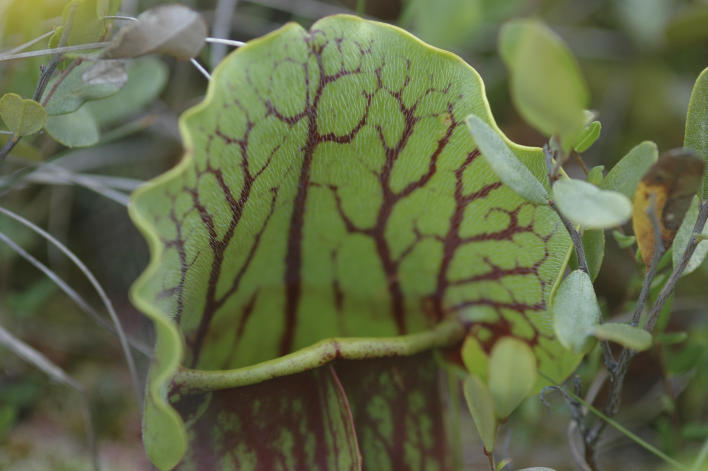
*Sarracenia purpurea.* (Photo: Nicholas J. Gotelli)

Gotelli and Ellison counted all the pitcher plant residents through the course of an entire field season in which the treatments were applied to the plants. They next evaluated how well the different models—incorporating different assumptions about habitat, keystone species, and food-web interactions—predicted the observed abundances. Overall, food-web models provided more-accurate indicators of species abundance than simple single-factor models, in which the abundance of each species depends on only one variable. The model based on habitat size alone (that is, the water volume), for example, did not do a good job of predicting individual species’ abundances, undercutting the traditional notion that habitat contraction leads to a simple decline in abundance across the board.

The best predictors of abundance were models that incorporated trophic structure—including the mosquito keystone model. This model accurately reflected the pitcher plant food web, with mosquito larvae preying on rotifers, and sarcophagid flies preying on mosquito larvae. “Bottom-up” food-web models (in which links flow from prey to predator) predicted that changes in bacteria population size influence protozoa abundances, which in turn affect mosquito numbers, and that changes in bacteria abundance also affect mite numbers, which impact rotifer abundance. This scenario lends support to the model of a *Sarracenia* food web in which each link in the chain performs a specialized service in breaking down the arthropod prey that is used by the next species in the processing chain.

With over 200 million acres of the world’s forestlands destroyed in the 1990s alone, and an estimated 40% increase in the human population by 2050, a growing number of species will be forced to cope with shrinking habitat. Instead of trying to determine how individual species might respond to habitat loss, Gotelli and Ellison argue that incorporating trophic structure into ecological models may yield more-accurate predictions of species abundance—a critical component of species restoration strategies.

